# Association between waist circumference or weight change after smoking cessation and incidence of cardiovascular disease or all-cause death in Korean adults with type 2 diabetes

**DOI:** 10.3389/fendo.2024.1493663

**Published:** 2024-11-28

**Authors:** Heajung Lee, Jaeyong Shin, Jae Woo Choi

**Affiliations:** ^1^ Department of Statistics and Data Science, Yonsei University, Seoul, Republic of Korea; ^2^ Department of Preventive Medicine, Yonsei University, Seoul, Republic of Korea; ^3^ Health Insurance Research Institute, National Health Insurance Service, Wonju, Republic of Korea

**Keywords:** cardiovascular disease, mortality, smoking cessation, waist change, weight change

## Abstract

**Objective:**

To investigate the association among smoking cessation, weight or waist circumference change post-cessation, and cardiovascular disease (CVD) or all-cause death among patients with type 2 Diabetes (T2D).

**Materials and methods:**

This retrospective cohort study included 32,142 patients with T2D classified according to changes in smoking status, post-cessation weight, and waist circumference. Especially for recent or long-term quitters, participants who changed from current to none/former smoker or from non-smoker to former smoker were defined as recent quitters, and those who changed from former to none/former smoker were defined as long-term quitters. CVD or all-cause death risk was evaluated.

**Results:**

A total of 5,845 participants were newly diagnosed with CVD, and 3,723 died during follow-up. After adjusting for potential confounding factors, compared with current smokers, the hazard ratios (HRs) for CVD were 0.94 (95% confidence interval [CI]: 0.85–1.03), 0.82 (95% CI: 0.74–0.90), and 0.82 (95% CI: 0.75–0.90) for recent quitters, long-term quitters, non-smokers, respectively; 0.88 (95% CI: 0.78–0.99), 0.68 (95% CI: 0.57–0.81), and 0.82 (95% CI: 0.67–1.00) for long-term quitters with no waist circumference gain, long-term quitters with waist circumference gain of 0.1–5.0 cm, and long-term quitters with waist circumference gain ≥5.0 cm, respectively; and 0.79 (95% CI: 0.71–0.89), 0.85 (95% CI: 0.74–0.98), and 0.84 (95% CI: 0.60–1.17) for long-term quitters with no weight gain, long-term quitters with weight gain of 2–5 kg, and long-term quitters with weight gain ≥5 kg, respectively. Similar associations were observed for all-cause death.

**Conclusions:**

Patients with T2D should maintain their weight and waist circumference after long-term smoking cessation to prevent CVD. It is more important for them to maintain weight rather than waist circumference to prevent all-cause death.

## Introduction

Cardiovascular disease (CVD) is a major complication in individuals with type 2 diabetes, accounting for approximately 90% of all diabetes types (1). Worldwide, the prevalence of CVD in individuals with type 2 diabetes is approximately 35% (2). A previous study revealed that the incidence of CVD in individuals with type 2 diabetes is approximately 2–3 times higher than that in those without (1). Because CVD is the leading cause of death in patients with diabetes and is considered a danger to human health (3–5), preventing the occurrence of cardiovascular events has become an important global issue.

Several lifestyle modifiable behaviors, such as diet control or exercise, have been suggested to prevent CVD in individuals with type 2 diabetes (3, 6). Among several lifestyle modification factors, smoking cessation is an important in preventing CVD in individuals with type 2 diabetes (1). The higher risk of CVD or death in current smokers with type 2 diabetes than in non-smokers has been consistently suggested in previous studies (1, 5, 7–11), and this finding is attributable to cigarette-induced biochemical action toward accelerating the progress of atherosclerosis (12). Regarding smoking cessation, a lower risk of CVD or death among quitters with type 2 diabetes than in current smokers has been reported in previous studies (8, 13, 14). However, only a few studies have investigated the association between smoking cessation and the incidence of CVD or death by stratifying quitters by smoking cessation duration (short-term or long-term).

Smoking cessation has a preventative association with CVD or death in individuals with type 2 diabetes, but at the same time, smoking cessation is associated with increase in the values of metabolic health indicators such as weight or waist circumference. Previous studies have reported that individuals who quit smoking experience significant weight gain after smoking cessation, compared with those who continue smoking (15–17). Similarly, a significant increase in waist circumference has been observed in quitters compared with continuous smokers (17, 18). The major cause of weight or waist circumference gain among quitters is the absence of the regulatory effect of nicotine on appetite and food intake, after smoking cessation (16, 19).

Two previous studies were conducted on the overall effect of smoking cessation and post-cessation weight or waist circumference gain on the incidence of CVD or death among patients with type 2 diabetes (20, 21). However, one study was focused on postmenopausal women recruited in the 90s, and smoking cessation duration was not considered in the analysis. Participants in the other cohort study mostly comprised Caucasians, and the association between post-cessation weight gain and CVD or death was examined based on each participant’s self-reported weight. Considering the differences in weight or waist circumference gain between Asian and non-Asian populations (22–25), it is necessary to explore how weight or waist circumference gain after smoking cessation among quitters is associated with the risk of incident CVD or death, particularly among Asian populations.

Therefore, the aim of this study was to investigate the risk of incident CVD or all-cause death among quitters with type 2 diabetes by distinguishing between short- and long-term quitters, using large-scale data of Asian populations, as well as to examine the risk of incident CVD or all-cause death among quitters with type 2 diabetes, considering post-cessation weight or waist circumference, by stratifying quitters by range of weight or waist circumference gain.

## Materials and methods

### Data source and study population

This retrospective cohort study used National Health Insurance System-National Sample Cohort (NHIS-NSC) database established by the National Health Insurance System in South Korea. NHIS-NSC database contains sociodemographic characteristics and medical information of about one million Koreans (26). A sample cohort included in NHIS-NSC database was sampled based on a stratified random sampling method, and followed up to 2019 (27). It includes qualification data, medical records such as diagnoses based on the International Statistical Classification of Diseases and Related Health Problems 10th revision (ICD-10) or medical procedures, and drug prescriptions. This study used NHIS-NSC dataset from 2002 to 2019.

**Figure 1:**
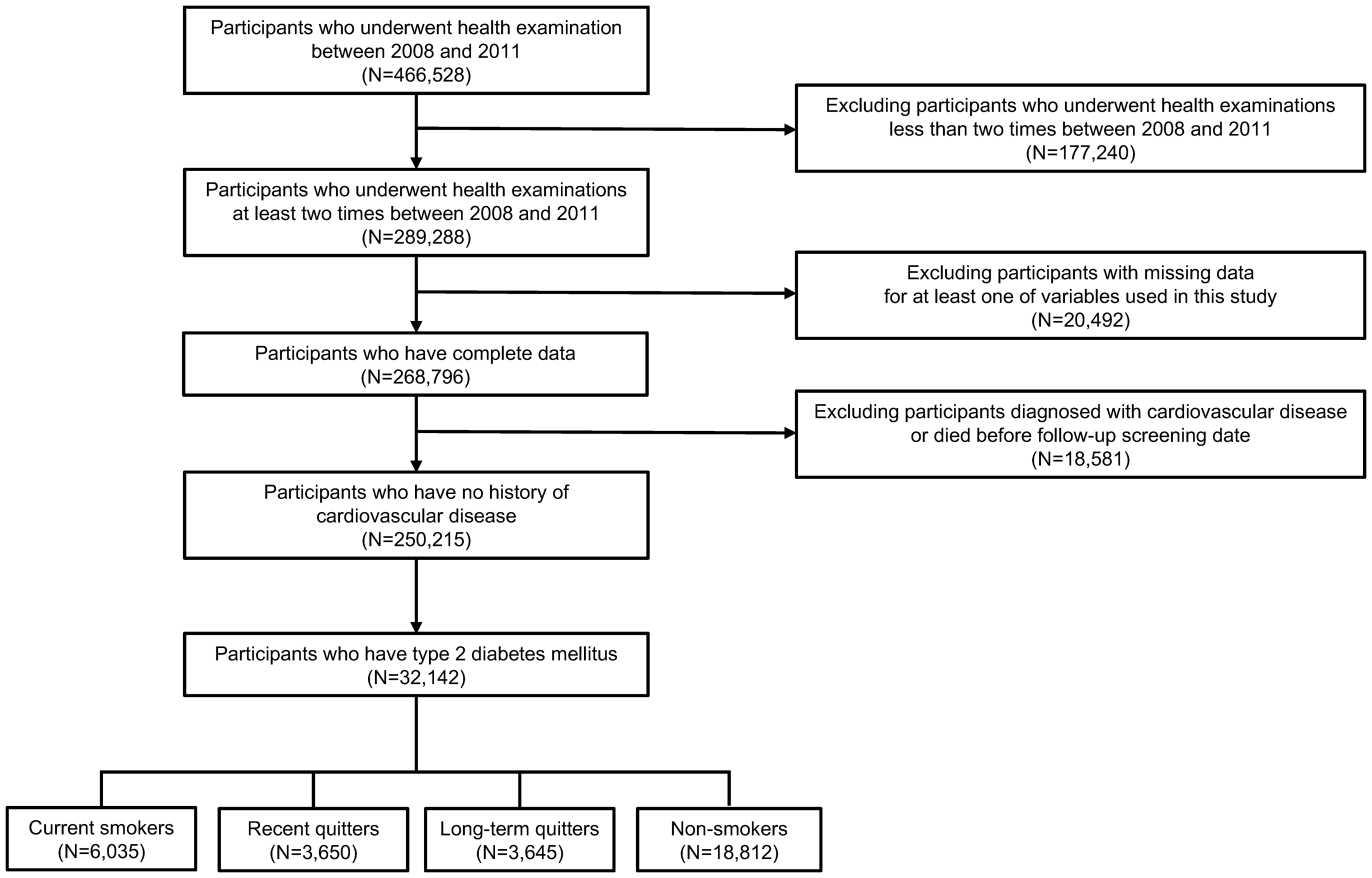
Flowchart of study participants. Participants were selected from National Health Insurance System-National Sample Cohort dataset. Of initial 466,528 participants, ineligible participants were excluded, resulting in a final sample of 32,142 participants.

Among 1,021,208 participants in NHIS-NSC dataset, we first selected participants who underwent medical health examinations at least once between 2008 and 2011 considering that data on waist circumference has been available since 2008 (28). Among 466,528 participants who received health examinations at least one time between 2008 and 2011, we excluded 177,240 participants who had only one time of medical check-up between 2008 and 2011, since at least two time of examination records is needed to capture a participant’s change of waist circumference, weight and smoking status. During 4-year of screening period, the first health examination date was defined as a participant’s first screening date, and the last health examination date was defined as a participant’s follow-up screening date. Next, we excluded 20,492 participants with missing data for at least one of measurements used in this study. To minimize possibility of reverse causality between waist or weight change after smoking cessation and the incidence of CVD or all-cause death, 18,581 participants who were diagnosed with CVD before follow-up screening date were also excluded. Lastly, we only included participants with type 2 diabetes, who satisfy any of following criteria: (i) fasting serum glucose concentration ≥ 126 mg/dL, or (ii) at least two claims of ICD-10 code (E11-E14) with the prescription of anti-diabetic agents before follow-up screening date (29). Finally, 32,142 participants were included in this study, as represented in **Figure 1**.

### Study outcome and follow-up

Study outcome was the incidence of CVD or all-cause death. The incidence of CVD was defined as follows: (i) at least two claims of ICD-10 code (I20–I25) for ischemic heart disease, or (ii) at least two claims of ICD-10 (I60–I64) for cerebrovascular disease after follow-up screening date (30). The occurrence of all-cause death was defined based on the presence of a participant’s date of death caused by any reason. Follow-up end date for CVD or all-cause death was defined as the earliest date of following three dates: (i) first incidence date of CVD (ii) date of death, or (iii) study end date (December 31, 2019).

### Change in waist circumference, weight, and smoking status

Based on change from a patient’s self-reported smoking status at first screening date to that at follow-up screening date, participants were categorized into four groups as follows: (i) change from any none/former/current smoker to current smoker (current smoker), (ii) change from current smoker to none/former smoker, or change from non-smoker to former smoker (recent quitter), (iii) change from former smoker to none/former smoker (long-term quitter), and (iv) change from non-smoker to non-smoker (non-smoker) (24).

Waist circumference was measured as the halfway between the lowest rib cage and top of the iliac crest in an upright position, and recorded in millimeter (31). Based on absolute change in waist circumference, defined as waist circumference at first screening date subtracted from that at follow-up screening date, participants were categorized into three groups as follows: <0.1 cm (no waist gain), 0.1–5.0 cm, and ≥5.0 cm. Cut-off points for waist circumference were set based on commonly used cut-off points, or incremental units to segment participants according to waist circumference in previous studies (32–35). Weight was measured using an electronic scale while a participant was dressed in simple clothes, and recorded as a valid integer value in kilograms (36). In line with waist circumference, change in weight was computed as weight at follow-up screening date minus that at first screening date, and utilized to categorize participants into three groups as follows: <2 kg (no weight gain), 2–5 kg, and ≥5 kg. Cut-off points for weight was determined based on previous studies on smoking or weight among Asian population (37, 38).

In order to investigate the risk for CVD or all-cause death in association with waist gain after smoking cessation, participants were classified into one of eight groups as follows: current smoker, recent quitter with no waist gain, recent quitter with waist gain of 0.1-5.0 cm, recent quitter with waist gain of ≥5.0 cm, long-term quitter with no waist gain, long-term quitter with waist gain of 0.1-5.0 cm, long-term quitter with waist gain of ≥5.0 cm, and non-smoker. For the purpose of exploring the risk for CVD or all-cause death in association with weight gain after smoking cessation, participants were classified into one of eight groups as follows: current smoker, recent quitter with no weight gain, recent quitter with weight gain of 2-5 kg, recent quitter with weight gain of ≥5 kg, long-term quitter with no weight gain, long-term quitter with weight gain of 2-5 kg, long-term quitter with weight gain of ≥5 kg, and non-smoker.

### Covariates

To minimize confounding effects by nuisance variables, we considered following variables as covariates for adjustment: age, sex, household income level, systolic blood pressure, diastolic blood pressure, total cholesterol, alcohol consumption, physical activity, comorbidities at follow-up screening date, and body mass index (BMI) at first screening date. Household income level was categorized as follows: <40^th^ percentile (low), 40^th^-80^th^ percentile (middle), and 80^th^-100^th^ percentile (high). BMI was calculated as a participant’s weight in kilograms divided by the square of the participant’s height in meters, and categorized into following five groups according to World Health Organization’s classification guide for Asians: <18.5 kg/m^2^ (underweight), 18.5–23.0 kg/m^2^ (normal weight), 23.0–25.0 kg/m^2^ (overweight), 25.0–30.0 kg/m^2^ (class I obese), and ≥30 kg/m^2^ (class II obese) (39). Total cholesterol was measured by the enzymatic method after drawing an examinee’s blood with a disposable syringe or vacuum test tube including a disposable needle when the examinee was in fasting state for at least 12 hours before examination (40). Alcohol consumption was classified into three groups according to the average number of drinking days weekly as follows: <1 (none), 1–4 (light-to-moderate), ≥5 (heavy). Physical activity was classified into three groups according to the average number of days of exercise weekly as follows: <1 (none), 1–4 (light-to-moderate), ≥5 (vigorous).

We investigated whether a participant has history of hypertension, dyslipidemia, or cancer, which are representative comorbidities among patients with type 2 diabetes (41). Hypertension was defined according to the following criteria: (i) systolic/diastolic blood pressure ≥ 140/90 mmHg, or (ii) at least two claims of ICD-10 code (I10-I15) with the prescription of anti-hypertensive agents. Dyslipidemia was defined according to the following criteria: (i) total cholesterol level ≥ 240 mg/dL, or (ii) at least two claims of ICD-10 code (E78) with the prescription of lipid-lowering agents. Cancer was defined as using at least two claims of ICD-10 code (C00–C97).

### Statistical analysis

Descriptive statistics were calculated for numerical or categorical variables included in this study. While numerical variables were summarized as mean with standard deviation, categorical variables were summarized as number of cases with proportion in percentage. In order to find statistical difference in a variable among groups, ANOVA test was used for numerical variables, and Chi-square test was used for categorical variables. We calculated hazard ratio (HR) and corresponding 95% confidence interval (CI) using Cox proportional hazards model (42) to compare the risk of CVD or all-cause death among different smoking cessation groups, or among different smoking groups in combination with post-cessation weight or waist change. Unadjusted HR was calculated from univariate Cox model. Adjusted HR was calculated from hierarchically adjusted Cox models as follows: (i) Model 1 (sex and age adjusted), (ii) Model 2 (Model 1+baseline BMI, household income level, systolic blood pressure, diastolic blood pressure, total cholesterol, alcohol consumption, physical activity, hypertension, dyslipidemia, cancer adjusted). The proportionality assumption in hazards over time was checked by score test, which proved the appropriateness of this assumption in result. All analysis was performed using SAS software (version 9.4, SAS Institute, Cary, NC, USA) and R version 4.2.3 (R Foundation for Statistical Computing, Vienna, Austria; http://www.R-project.org). A two-sided p-value of less than 0.05 was considered statistically significant.

## Results

### Baseline characteristics

Of 32,142 eligible participants, 5,845 participants were newly diagnosed with CVD during a median follow-up of 8.42 years (interquartile range: 8.00–9.00), and 3,723 participants died during a median follow-up of 8.17 years (interquartile range: 8.17–9.08). **Table 1** represents the baseline characteristics of study participants according to change in smoking status. In terms of all variables in **Table 1**, participants tended to have significantly different mean or proportion according to change in smoking status. Compared to current smokers, recent or long-term quitters were older, had higher household income, were more likely to be overweight at baseline, drank less, worked out more frequently in a week, and were more likely to have a history of comorbidities. Both systolic and diastolic blood pressure were higher in recent or long-term quitters than those for current smokers.

**Table 1:** Baseline characteristics of study participants according to change in smoking status.

Variable	All	Change in smoking status				*p*-value
		Current smokers	Recent quitters	Long-term quitters	Non-smokers	
Number of participants	*32,142*	6,035 (18.8)	3,650 (11.4)	3,645 (11.3)	18,812 (58.5)	<.001
Sex						<.001
Male	16,782 (52.2)	5,658 (93.8)	3,406 (93.3)	3,482 (95.5)	4,236 (22.5)	
Female	15,360 (47.8)	377 (6.3)	244 (6.7)	163 (4.5)	14,576 (77.5)	
Age, years	58.9 ± 11.7	54.3 ± 11.6	58.8 ± 11.3	59.4 ± 10.9	60.3 ± 11.5	<.001
Baseline body mass index (kg/m^2^)						<.001
<18.5	674 (2.1)	141 (2.3)	62 (1.7)	50 (1.4)	421 (2.2)	
18.5-23	9,793 (30.5)	1,869 (31.0)	983 (26.9)	938 (25.7)	6,003 (31.9)	
23-25	8,449 (26.3)	1,640 (27.2)	1,028 (28.2)	994 (27.3)	4,787 (25.5)	
25-30	11,594 (36.1)	2,090 (34.6)	1,438 (39.4)	1,494 (41.0)	6,572 (34.9)	
≥30	1,632 (5.1)	295 (4.9)	139 (3.8)	169 (4.6)	1,029 (5.5)	
Total cholesterol (mg/dL)	194.2 ± 42.1	193.4 ± 38.9	190.7 ± 55.8	189.3 ± 38.0	196.1 ± 40.7	<.001
Systolic blood pressure (mmHg)	126.4 ± 15.2	125.7 ± 14.7	127.0 ± 14.4	127.9 ± 14.5	126.2 ± 15.7	<.001
Diastolic blood pressure (mmHg)	77.4 ± 9.8	78.0 ± 9.7	78.2 ± 9.7	78.4 ± 9.6	76.8 ± 9.8	<.001
Household income						<.001
Low	5,052 (15.7)	927 (15.4)	521 (14.3)	462 (12.7)	3,142 (16.7)	
Middle	13,303 (41.4)	2,700 (44.7)	1,423 (39.0)	1,389 (38.1)	7,791 (41.4)	
High	13,787 (42.9)	2,408 (39.9)	1,706 (46.7)	1,794 (49.2)	7,879 (41.9)	
Alcohol consumption (days/week)						<.001
<1	20,068 (62.4)	1,852 (30.7)	1,616 (44.3)	1,469 (40.3)	15,131 (80.4)	
1-4	10,385 (32.3)	3,550 (58.8)	1,708 (46.8)	1,835 (50.3)	3,292 (17.5)	
≥5	1,689 (5.3)	633 (10.5)	326 (8.9)	341 (9.4)	389 (2.1)	
Physical activity (days/week)						<.001
<1	19,875 (61.8)	3,397 (56.3)	1,973 (54.1)	1,840 (50.5)	12,665 (67.3)	
1-4	9,761 (30.4)	2,247 (37.2)	1,336 (36.6)	1,399 (38.4)	4,779 (25.4)	
≥5	2,506 (7.8)	391 (6.5)	341 (9.3)	406 (11.1)	1,368 (7.3)	
Comorbidity						
Hypertension	16,457 (51.2)	2,633 (43.6)	1,893 (51.9)	2,002 (54.9)	9,929 (52.8)	<.001
Dyslipidemia	11,346 (35.3)	2,000 (33.1)	1,202 (32.9)	1,329 (36.5)	6,815 (36.2)	<.001
Cancer	1,308 (4.1)	140 (2.3)	195 (5.3)	207 (5.7)	766 (4.1)	<.001

p-value represents statistical difference in basic characteristics among study participants according to change in smoking status. p-values are calculated by ANOVA test, or Chi-square test. Data are expressed as the mean±standard deviation, or number of participants (%). Italicized value means total number of study participants in our study.

### Association between smoking cessation and CVD or all-cause death

**Table 2** represents HR and 95% CI for the incidence of CVD or all-cause death associated with change in smoking status. After adjusting for possible confounding factors, HR for CVD were 0.94 (95% CI: 0.85–1.03; p = 0.192), 0.82 (95% CI: 0.74–0.90; p <.001), and 0.82 (95% CI: 0.75–0.90; p <.001) for recent quitters, long-term quitters, non-smokers compared to current smokers, respectively. Long-term quitters or non-smokers had significantly lower risk for CVD, compared to current smokers. In case of all-cause death, fully adjusted HR for all-cause death was 0.67 (95% CI: 0.60–0.75; p <.001), 0.62 (95% CI: 0.56–0.70; p <.001), and 0.55 (95% CI: 0.50–0.61; p <.001) for recent quitters, long-term quitters, non-smokers compared to current smokers, respectively. Compared to current smokers, the risk for all-cause death was significantly lower in all of three groups (recent quitters, long-term quitters, non-smokers).

**Table 2:** Association between change in smoking status and the risk of cardiovascular disease and mortality.

Outcome	Change in smoking status	Number of study participants	Event	Person-years	Incidence rates (per 1,000 person-years)	Unadjusted Model		Adjusted Model 1		Adjusted Model 2	
						HR (95% CI)	*p*-value	HR (95% CI)	*p*-value	HR (95% CI)	*p*-value
Cardiovascular disease	Current smokers	6,035	1,003	45,743	21.9	1.00		1.00		1.00	
	Recent quitters	3,650	706	27,418	25.7	1.18 (1.07, 1.29)	<.001	0.96 (0.87, 1.06)	0.397	0.94 (0.85, 1.03)	0.192
	Long-term quitters	3,645	649	27,553	23.6	1.07 (0.97, 1.19)	0.156	0.85 (0.77, 0.94)	0.002	0.82 (0.74, 0.90)	<.001
	Non-smokers	18,812	3,487	145,693	23.9	1.09 (1.02, 1.17)	0.015	0.87 (0.79, 0.94)	<.001	0.82 (0.75, 0.90)	<.001
Mortality	Current smokers	6,035	836	49,895	16.8	1.00		1.00		1.00	
	Recent quitters	3,650	523	30,324	17.2	1.02 (0.91, 1.14)	0.745	0.67 (0.60, 0.75)	<.001	0.67 (0.60, 0.75)	<.001
	Long-term quitters	3,645	478	30,276	15.8	0.96 (0.86, 1.07)	0.467	0.62 (0.55, 0.69)	<.001	0.62 (0.56, 0.70)	<.001
	Non-smokers	18,812	1,886	160,562	11.7	0.67 (0.62, 0.73)	<.001	0.55 (0.50, 0.61)	<.001	0.55 (0.50, 0.61)	<.001

Model 1: adjusted for sex, and age; Model 2: adjusted for sex, age, baseline body mass index, systolic blood pressure, diastolic blood pressure, total cholesterol, household income, alcohol consumption, physical activity, hypertension, dyslipidemia, and cancer.

CI, confidence interval; HR, hazard ratio.

### Association between waist gain after smoking cessation and CVD or all-cause death

**Figure 2:**
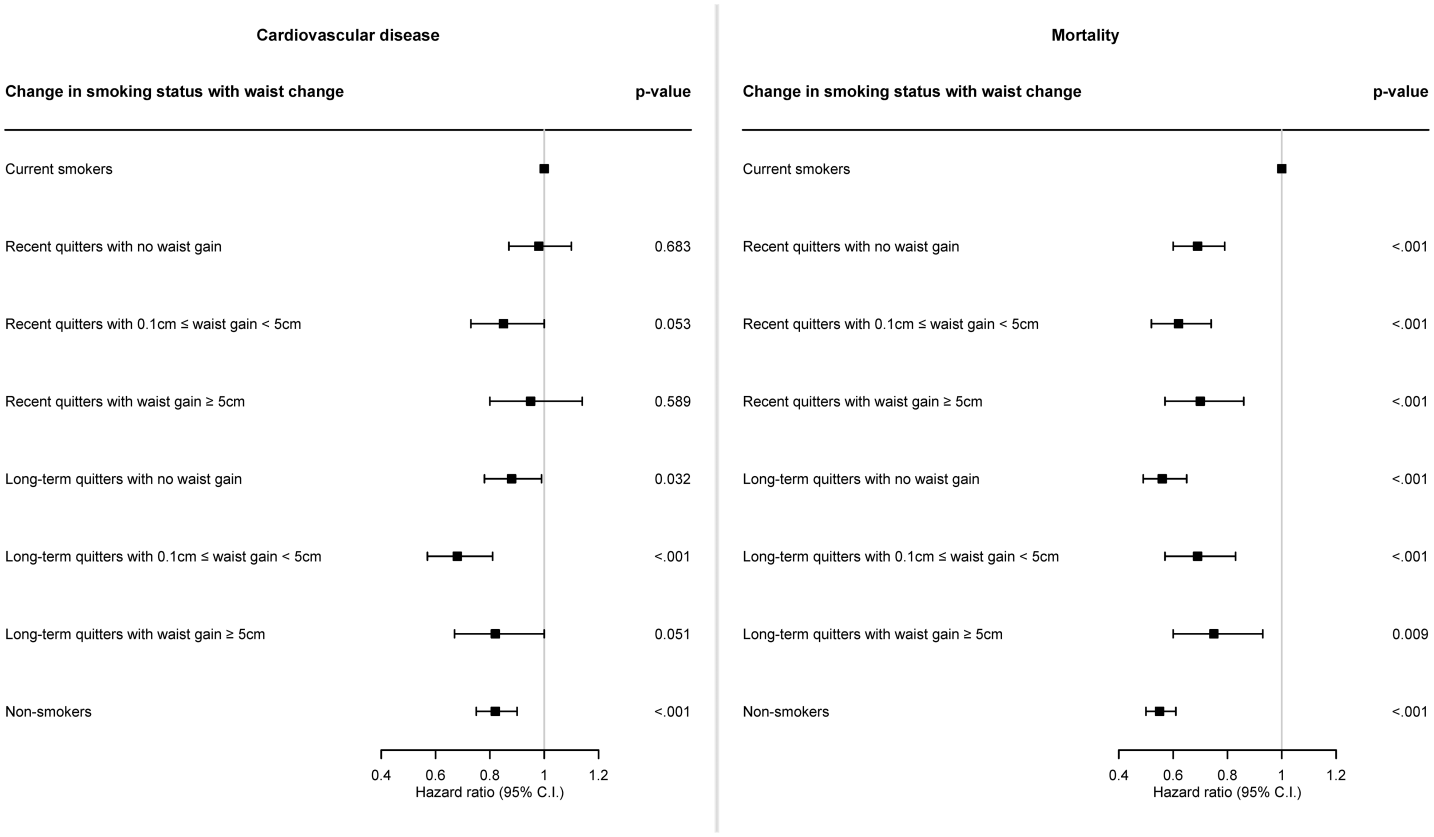
Forest plot of hazard ratio for the risk of cardiovascular disease and mortality according to waist gain after smoking cessation. Cox proportional hazards model was constructed to examine the risk of cardiovascular disease and mortality according to waist gain after smoking cessation. Hazard ratio was calculated adjusting for sex, age, baseline body mass index, household income level, systolic blood pressure, diastolic blood pressure, total cholesterol, alcohol consumption, physical activity, hypertension, dyslipidemia, cancer.

**Figure 2** graphically shows HR and 95% CI for the incidence of CVD or all-cause death associated with waist gain after smoking cessation, and corresponding results are also described in **Supplementary Table 1**. In case of recent quitters, fully adjusted HR for CVD was 0.98 (95% CI: 0.87–1.10; p = 0.683), 0.85 (95% CI: 0.73–1.00; p = 0.053), and 0.95 (95% CI: 0.80–1.14; p = 0.589) for recent quitters with no waist gain, recent quitters with waist gain of 0.1-5.0 cm, and recent quitters with waist gain of ≥5.0 cm, compared to current smokers, respectively. In case of long-term quitters, fully adjusted HR for CVD was 0.88 (95% CI: 0.78–0.99; p = 0.032), 0.68 (95% CI: 0.57–0.81; p <.001), and 0.82 (95% CI: 0.67–1.00; p = 0.051) for long-term quitters with no waist gain, long-term quitters with waist gain of 0.1-5.0 cm, and long-term quitters with waist gain of ≥5.0 cm, compared to current smokers, respectively. Compared to current smokers, HR for CVD was 0.82 (95% CI: 0.75–0.90; p <.001) for non-smokers.

In terms of all-cause death, fully adjusted HR for all-cause death was 0.69 (95% CI: 0.60–0.79; p <.001), 0.62 (95% CI: 0.52–0.74; p <.001), and 0.70 (95% CI: 0.57–0.86; p <.001) for recent quitters with no waist gain, recent quitters with waist gain of 0.1-5.0 cm, and recent quitters with waist gain of ≥5.0 cm, compared to current smokers, respectively. In case of long-term quitters, fully adjusted HR for all-cause death was 0.56 (95% CI: 0.49–0.65; p <.001), 0.69 (95% CI: 0.57–0.83; p <.001), and 0.75 (95% CI: 0.60–0.93; p = 0.009) for long-term quitters with no waist gain, long-term quitters with waist gain of 0.1-5.0 cm, and long-term quitters with waist gain of ≥5.0 cm, compared to current smokers, respectively. Compared to current smokers, HR for all-cause death was 0.55 (95% CI: 0.50–0.61; p <.001) for non-smokers.

### Association between weight gain after smoking cessation and CVD or all-cause death

**Figure 3:**
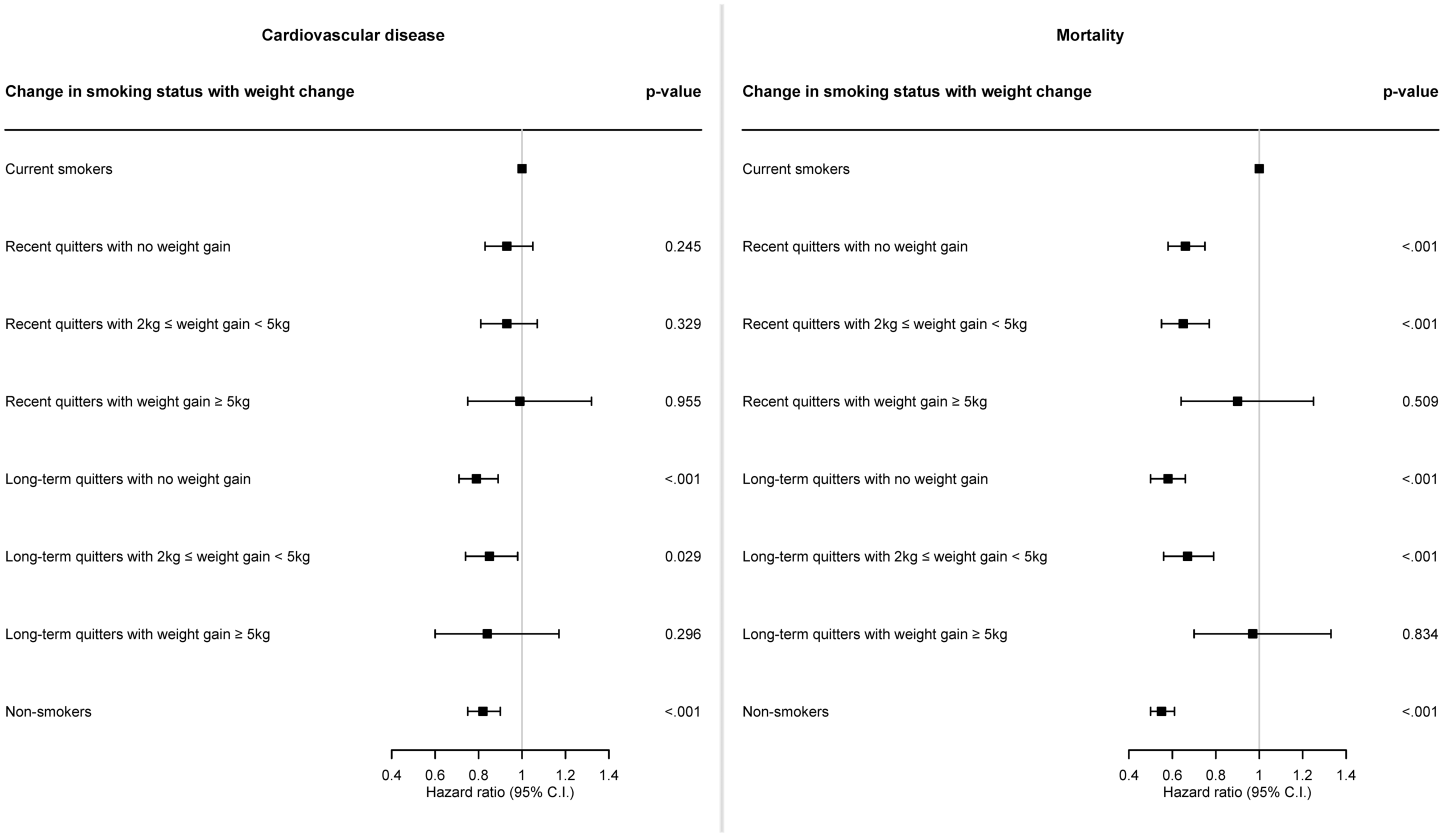
Forest plot of hazard ratio for the risk of cardiovascular disease and mortality according to weight gain after smoking cessation. Cox proportional hazards model was constructed to examine the risk of cardiovascular disease and mortality according to weight gain after smoking cessation. Hazard ratio was calculated adjusting for sex, age, baseline body mass index, household income level, systolic blood pressure, diastolic blood pressure, total cholesterol, alcohol consumption, physical activity, hypertension, dyslipidemia, cancer.

**Figure 3** is the forest plot representing HR and 95% CI for the incidence of CVD or all-cause death associated with weight gain after smoking cessation, and corresponding results are also described in **Supplementary Table 2**. In case of recent quitters, fully adjusted HR for CVD was 0.93 (95% CI: 0.83–1.05; p = 0.245), 0.93 (95% CI: 0.81–1.07; p = 0.329), and 0.99 (95% CI: 0.75–1.32; p = 0.955) for recent quitters with no weight gain, recent quitters with weight gain of 2-5 kg, and recent quitters with weight gain of ≥5 kg, compared to current smokers, respectively. In case of long-term quitters, fully adjusted HR for CVD was 0.79 (95% CI: 0.71–0.89; p <.001), 0.85 (95% CI: 0.74–0.98; p = 0.029), and 0.84 (95% CI: 0.60–1.17; p = 0.296) for long-term quitters with no weight gain, long-term quitters with weight gain of 2-5 kg, and long-term quitters with weight gain of ≥5 kg, compared to current smokers, respectively. Compared to current smokers, HR for CVD was 0.82 (95% CI: 0.75–0.90; p <.001) for non-smokers.

In terms of all-cause death, fully adjusted HR for all-cause death was 0.66 (95% CI: 0.58–0.75; p <.001), 0.65 (95% CI: 0.55–0.77; p <.001), and 0.90 (95% CI: 0.64–1.25; p = 0.509) for recent quitters with no weight gain, recent quitters with weight gain of 2-5 kg, and recent quitters with weight gain of ≥5 kg, compared to current smokers, respectively. In case of long-term quitters, fully adjusted HR for all-cause death was 0.58 (95% CI: 0.50–0.66; p <.001), 0.67 (95% CI: 0.56–0.79; p <.001), and 0.97 (95% CI: 0.70–1.33; p = 0.834) for long-term quitters with no weight gain, long-term quitters with weight gain of 2-5 kg, and long-term quitters with weight gain of ≥5 kg, compared to current smokers, respectively. Compared to current smokers, HR for all-cause death was 0.55 (95% CI: 0.50–0.61; p <.001) for non-smokers.

## Discussion

We examined the association between smoking cessation and the risk of CVD or all-cause death among patients with type 2 diabetes. The risk of CVD among long-term quitters and non-smokers was significantly lower than that among current smokers. However, there was no significant association between recent quitting and the risk of CVD. The risk of all-cause death was significantly lower among recent quitters, long-term quitters, and non-smokers, compared with current smokers. In addition, we examined the association between weight or waist circumference gain after smoking cessation and the risk of CVD or all-cause death among patients with type 2 diabetes. Long-term quitters with no weight/waist circumference gain or <5 kg/5.0 cm weight/waist circumference gain had a significantly lower risk of CVD than did current smokers. The risk of all-cause death was significantly lower among both short- and long-term quitters than among current smokers, regardless of waist circumference gain after smoking cessation. However, regarding weight change, the risk of all-cause death was significantly lower among short- or long-term quitters with no weight gain or <5 kg weight gain than among current smokers.

Several previous studies have reported that long-term smoking cessation is associated with low risk of CVD among patients with type 2 diabetes (20, 21, 43), consistent with our results for long-term quitters. Although a previous study reported that long-term quitters with type 2 diabetes had a lower but insignificant risk of CVD than did current smokers (13), it should be noted that there was insufficient statistical power for this result due to the extremely small number of CVD events among recent quitters. The risk of all-cause death was lower among recent quitters, long-term quitters, and non-smokers than in current smokers. This result is consistent with those of previous studies on the association between smoking cessation and the risk of all-cause death in patients with type 2 diabetes (7, 8, 20).

Regarding weight gain among quitters, the estimated risk of CVD was lower among recent quitters, regardless of how much weight patients with type 2 diabetes gained after smoking cessation, compared with current smokers; however, this estimate was not statistically significant. This insignificant association between short-term smoking cessation and the risk of CVD was consistent with the results of a previous study that included only patients with diabetes (13) and studies that included both patients with and without (44). Long-term quitters with no weight gain or less than 5 kg weight gain had a significantly lower risk of CVD than current smokers. However, there was no significant association between weight gain >5 kg after long-term smoking cessation and the risk of CVD. Similar to our results, a previous study on postmenopausal women revealed that long-term quitters who gained <5 kg had a significantly lower risk of coronary heart disease, but long-term quitters who gained >5 kg had no significant association with the risk of coronary heart disease, compared with the corresponding current smokers (21). One previous study revealed a significantly lower risk of CVD-related death among long-term quitters who gained >5 kg within 2–6 years after smoking cessation, compared with current smokers (20); however, this inconsistency with our results may be due to differences in study designs, such as study population (Asian vs. non-Asian population) or outcome (CVD vs. CVD-related death).

Regarding waist circumference gain among quitters, the estimated risk of CVD was lower among recent quitters, regardless of how much waist circumference patients with type 2 diabetes gained after smoking cessation, compared with current smokers; however, this estimate was not statistically significant. Long-term quitters with no waist circumference gain or <5.0 cm waist circumference gain had a significantly lower risk of CVD than current smokers. However, there was no significant association between gaining >5.0 cm after long-term smoking cessation and the risk of CVD. This result is in line with the non-significant association between weight gain >5 kg weight gain after long-term smoking cessation and CVD risk. Since waist circumference gain >5.0 cm can attenuate the effect of long-term smoking cessation on lowering the risk of CVD, long-term quitters with diabetes should be cautioned against excessive waist circumference gain after smoking cessation to expect a significant effect of long-term smoking cessation on lowering the risk of CVD.

Both recent and long-term quitters had significantly lower risks of all-cause death than current smokers, regardless of how much waist circumference they gained after smoking cessation. Although our result was inconsistent with that of a previous study on male patients with type 2 diabetes, which revealed a lower but insignificant risk of all-cause death among quitters with BMI gain than among current smokers (45); however, this previous result is limited by its lack of statistical power due to the extremely small number of events. While quitters with no weight gain or <5 kg weight gain had a significantly lower risk of all-cause death than current smokers, there was no significant association between >5 kg weight gain after short or long-term smoking cessation and the risk of all-cause death. Previous studies have suggested a significant association between >5 kg of weight gain after smoking cessation and the risk of all-cause death (20, 44, 46); however, this inconsistency with our results may be caused by the heterogeneous nature of the study cohort, such as patients with/without diabetes or Asian/non-Asian population.

Although there were little studies that explore underlying biological mechanism contributing to difference in post-cessation weight change and post-cessation waist change in association with the risk of all-cause death, difference between overall obesity and central obesity related with smoking can be one possible evidence. While weight has been widely used as an indicator of overall fatness, waist circumference has been used as a marker of central obesity (47). In this regard, one previous study on type 2 diabetes patients reported that current smokers have significantly higher central obesity than non-smokers or former smokers, but there was no significant difference in overall obesity among patients with different smoking status (48). Similar results were found in previous studies including both patients with and without type 2 diabetes (49, 50). These previous results can imply difference in working mechanism between weight and waist change after smoking cessation, but further thorough research is needed to back up this evidence.

This study has several limitations. First, we excluded participants who were diagnosed with CVD or died before the follow-up screening date to minimize the possible effects of reverse causality. Nevertheless, the causal relationship between weight or waist circumference change after smoking cessation and the risk of incident CVD or all-cause death could not be determined because of the retrospective nature of this study. Second, since smoking-related information were self-reported, we cannot completely eliminate any possible effects of survey-based bias, such as recall bias, on our findings. Third, the start and end dates for smoking could not specified because of the unavailability of such information. In addition, it was impossible to capture changes in smoking status that may have occurred between the health examination dates. Future studies that include additional information on smoking status are recommended to precisely distinguish participants according to smoking status. Fourth, a smoker’s intensity of smoking was not reflected in our analysis due to data unavailability. If such data can be available, further studies on smokers classified according to not only smoking duration but also intensity of smoking, such as light or heavy smokers, are recommended. Fifth, we cannot differentiate between CVD-related death or non-CVD death since data on cause of death was not provided due to data sensitivity. Sixth, we determined cut-off points for weight or waist increment in light of previous studies on Asians, but evidence regarding representativeness of these cut-off points is insufficient. Hence, our findings need to be interpreted with caution, and we suggest further studies on optimal cut-off points for weight or waist increment. Finally, since Korean adults aged >18 years constituted most of our cohort, direct comparison or extrapolation of our findings to people of different ethnicities or regions requires special attention, and our findings cannot be generalized without logical reasons.

## Conclusion

In conclusion, we demonstrated that long-term smoking cessation is significantly associated with reduced CVD risk in patients with type 2 diabetes. We also demonstrated that long-term smoking cessation with no weight/waist circumference gain or <5 kg/5.0 cm weight/waist circumference gain is significantly associated with decreased CVD risk among patients with type 2 diabetes. Therefore, our findings suggest that adults with type 2 diabetes should maintain their weight or waist circumference after long-term smoking cessation to prevent CVD. In addition, both short-and long-term smoking cessation were significantly associated with reduced risk of all-cause death, irrespective of how much waist circumference patients with type 2 diabetes gained after smoking cessation. However, the decreased risk of all-cause death was significantly associated only with either short- or long-term smoking cessation, with no weight gain or <5 kg weight gain among patients with type 2 diabetes. Our findings suggest that long-term quitters with type 2 diabetes should pay more attention to maintaining their weight rather than waist circumference to prevent all-cause death.

## Data Availability

The data analyzed in this study is subject to the following licenses/restrictions: The data are available from the Korean National Health Insurance Service (NHIS), but access to confidential data is limited to researchers who meet the necessary criteria. Requests to access these datasets should be directed to https://nhiss.nhis.or.kr/en/z/a/001/lpza001m01en.do.
