# Supplementary material for: Association between waist circumference or weight change after smoking cessation and incidence of cardiovascular disease or all-cause death in Korean adults with type 2 diabetes
Source: Front Endocrinol (Lausanne). 2024 Nov 28;15:1493663. doi: 10.3389/fendo.2024.1493663 (PMC11634621; doi:10.3389/fendo.2024.1493663)
Supplement: Supplementary file 1 [file Table1.docx]

Supplementary Table 1. Association between waist gain after smoking cessation and the risk of cardiovascular disease and mortality

|  |  |  |  |  |  | Unadjusted Model | | Adjusted Model 1 | | Adjusted Model 2 | |
| --- | --- | --- | --- | --- | --- | --- | --- | --- | --- | --- | --- |
| Outcome | Change in smoking status  with waist gain | Number of  study participants | Event | Person-years | Incidence rates (per 1,000 person-years) | HR (95% CI) | *p*-value | HR (95% CI) | *p*-value | HR (95% CI) | *p*-value |
| Cardiovascular disease | Current smokers | 6,035 | 1,003 | 45,743 | 21.9 | 1.00 |  | 1.00 |  | 1.00 |  |
|  | Recent quitters  with no waist gain | 1,980 | 390 | 14,812 | 26.3 | 1.20  (1.07, 1.35) | 0.002 | 1.00  (0.89, 1.12) | 0.978 | 0.98  (0.87, 1.10) | 0.683 |
|  | Recent quitters  with 0.1cm≤waist gain<5cm | 1,002 | 180 | 7,655 | 23.5 | 1.07  (0.91, 1.26) | 0.394 | 0.86  (0.74, 1.01) | 0.072 | 0.85  (0.73, 1.00) | 0.053 |
|  | Recent quitters  with waist gain≥5cm | 668 | 136 | 4,951 | 27.5 | 1.26  (1.05, 1.50) | 0.013 | 0.99  (0.83, 1.19) | 0.920 | 0.95  (0.80, 1.14) | 0.589 |
|  | Long-term quitters  with no waist gain | 2,118 | 399 | 16,030 | 24.9 | 1.14  (1.01, 1.28) | 0.032 | 0.91  (0.81, 1.02) | 0.109 | 0.88  (0.78, 0.99) | 0.032 |
|  | Long-term quitters  with 0.1cm≤waist gain<5cm | 961 | 148 | 7,356 | 20.1 | 0.92  (0.77, 1.09) | 0.328 | 0.72  (0.60, 0.85) | <.001 | 0.68  (0.57, 0.81) | <.001 |
|  | Long-term quitters  with waist gain≥5cm | 566 | 102 | 4,168 | 24.5 | 1.12  (0.91, 1.37) | 0.293 | 0.87  (0.71, 1.07) | 0.192 | 0.82  (0.67, 1.00) | 0.051 |
|  | Non-smokers | 18,812 | 3,487 | 145,693 | 23.9 | 1.09  (1.02, 1.17) | 0.015 | 0.87  (0.79, 0.95) | <.001 | 0.82  (0.75, 0.90) | <.001 |
| Mortality | Current smokers | 6,035 | 836 | 49,895 | 16.8 | 1.00 |  | 1.00 |  | 1.00 |  |
|  | Recent quitters  with no waist gain | 1,980 | 281 | 16,447 | 17.1 | 1.01  (0.88, 1.15) | 0.944 | 0.70  (0.61, 0.80) | <.001 | 0.69  (0.60, 0.79) | <.001 |
|  | Recent quitters  with 0.1cm≤waist gain<5cm | 1,002 | 138 | 8,367 | 16.5 | 0.98  (0.82, 1.17) | 0.788 | 0.61  (0.51, 0.73) | <.001 | 0.62  (0.52, 0.74) | <.001 |
|  | Recent quitters  with waist gain≥5cm | 668 | 104 | 5,510 | 18.9 | 1.12  (0.92, 1.38) | 0.262 | 0.69  (0.56, 0.85) | <.001 | 0.70  (0.57, 0.86) | <.001 |
|  | Long-term quitters  with no waist gain | 2,118 | 248 | 17,692 | 14.0 | 0.85  (0.74, 0.98) | 0.024 | 0.56  (0.49, 0.65) | <.001 | 0.56  (0.49, 0.65) | <.001 |
|  | Long-term quitters  with 0.1cm≤waist gain<5cm | 961 | 138 | 7,945 | 17.4 | 1.06  (0.89, 1.28) | 0.497 | 0.66  (0.55, 0.79) | <.001 | 0.69  (0.57, 0.83) | <.001 |
|  | Long-term quitters  with waist gain≥5cm | 566 | 92 | 4,639 | 19.8 | 1.20  (0.97, 1.49) | 0.102 | 0.72  (0.58, 0.89) | 0.003 | 0.75  (0.60, 0.93) | 0.009 |
|  | Non-smokers | 18,812 | 1,886 | 160,562 | 11.7 | 0.67  (0.62, 0.73) | <.001 | 0.55  (0.50, 0.61) | <.001 | 0.55  (0.50, 0.61) | <.001 |

Model 1: adjusted for sex, and age; Model 2: adjusted for sex, age, baseline body mass index, systolic blood pressure, diastolic blood pressure, total cholesterol, household income, alcohol consumption, physical activity, hypertension, dyslipidemia, and cancer.

CI, confidence interval; HR, hazard ratio.

Supplementary Table 2. Association between weight gain after smoking cessation and the risk of cardiovascular disease and mortality

|  |  |  |  |  |  | Unadjusted Model | | Adjusted Model 1 | | Adjusted Model 2 | |
| --- | --- | --- | --- | --- | --- | --- | --- | --- | --- | --- | --- |
| Outcome | Change in smoking status  with waist gain | Number of  study participants | Event | Person-years | Incidence rates (per 1,000 person-years) | HR (95% CI) | *p*-value | HR (95% CI) | *p*-value | HR (95% CI) | *p*-value |
| Cardiovascular disease | Current smokers | 6,035 | 1,003 | 45,743 | 21.9 | 1.00 |  | 1.00 |  | 1.00 |  |
|  | Recent quitters  with no weight gain | 2,115 | 411 | 15,878 | 25.9 | 1.18  (1.05, 1.32) | 0.005 | 0.95  (0.85, 1.07) | 0.404 | 0.93  (0.83, 1.05) | 0.245 |
|  | Recent quitters  with 2kg≤weight gain<5kg | 1,264 | 244 | 9,500 | 25.7 | 1.17  (1.02, 1.35) | 0.027 | 0.96  (0.83, 1.10) | 0.530 | 0.93  (0.81, 1.07) | 0.329 |
|  | Recent quitters  with weight gain≥5kg | 271 | 51 | 2,040 | 25.0 | 1.14  (0.86, 1.51) | 0.358 | 1.03  (0.78, 1.37) | 0.817 | 0.99  (0.75, 1.32) | 0.955 |
|  | Long-term quitters  with no weight gain | 2,205 | 386 | 16,716 | 23.1 | 1.05  (0.94, 1.18) | 0.390 | 0.82  (0.73, 0.92) | <.001 | 0.79  (0.71, 0.89) | <.001 |
|  | Long-term quitters  with 2kg≤weight gain<5kg | 1,233 | 226 | 9,299 | 24.3 | 1.11  (0.96, 1.28) | 0.163 | 0.90  (0.78, 1.04) | 0.145 | 0.85  (0.74, 0.98) | 0.029 |
|  | Long-term quitters  with weight gain≥5kg | 207 | 37 | 1,538 | 24.1 | 1.10  (0.79, 1.52) | 0.577 | 0.92  (0.66, 1.28) | 0.622 | 0.84  (0.60, 1.17) | 0.296 |
|  | Non-smokers | 18,812 | 3,487 | 145,693 | 23.9 | 1.09  (1.02, 1.17) | 0.015 | 0.87  (0.79, 0.94) | <.001 | 0.82  (0.75, 0.90) | <.001 |
| Mortality | Current smokers | 6,035 | 836 | 49,895 | 16.8 | 1.00 |  | 1.00 |  | 1.00 |  |
|  | Recent quitters  with no weight gain | 2,115 | 312 | 17,534 | 17.8 | 1.05  (0.92, 1.20) | 0.468 | 0.68  (0.60, 0.78) | <.001 | 0.66  (0.58, 0.75) | <.001 |
|  | Recent quitters  with 2kg≤weight gain<5kg | 1,264 | 174 | 10,537 | 16.5 | 0.97  (0.82, 1.14) | 0.713 | 0.63  (0.53, 0.74) | <.001 | 0.65  (0.55, 0.77) | <.001 |
|  | Recent quitters  with weight gain≥5kg | 271 | 37 | 2,253 | 16.4 | 1.00  (0.72, 1.39) | 0.986 | 0.80  (0.57, 1.11) | 0.178 | 0.90  (0.64, 1.25) | 0.509 |
|  | Long-term quitters  with no weight gain | 2,205 | 281 | 18,315 | 15.3 | 0.93  (0.82, 1.07) | 0.315 | 0.59  (0.52, 0.68) | <.001 | 0.58  (0.50, 0.66) | <.001 |
|  | Long-term quitters  with 2kg≤weight gain<5kg | 1,233 | 157 | 10,287 | 15.3 | 0.93  (0.78, 1.10) | 0.376 | 0.61  (0.51, 0.72) | <.001 | 0.67  (0.56, 0.79) | <.001 |
|  | Long-term quitters  with weight gain≥5kg | 207 | 40 | 1,674 | 23.9 | 1.45  (1.05, 1.99) | 0.022 | 0.97  (0.71, 1.34) | 0.861 | 0.97  (0.70, 1.33) | 0.834 |
|  | Non-smokers | 18,812 | 1,886 | 160,562 | 11.7 | 0.67  (0.62, 0.73) | <.001 | 0.55  (0.50, 0.61) | <.001 | 0.55  (0.50, 0.61) | <.001 |

Model 1: adjusted for sex, and age; Model 2: adjusted for sex, age, baseline body mass index, systolic blood pressure, diastolic blood pressure, total cholesterol, household income, alcohol consumption, physical activity, hypertension, dyslipidemia, and cancer.

CI, confidence interval; HR, hazard ratio.
